# Prognostic value of hypoxia-responsive gene expression profile in patients diagnosed with head and neck squamous cell carcinoma

**DOI:** 10.1016/j.tranon.2023.101841

**Published:** 2023-11-27

**Authors:** Natasa Matic, Lina Pettersson, Felicia Sellebjerg, Lina Lindberg, Karin Roberg, Emilia Wiechec

**Affiliations:** a Department of Otorhinolaryngology in Linköping, Anaesthetics, Operations and Specialty Surgery Center, Region Östergötland, Linköping 58185, Sweden; b Department of Biomedical and Clinical Sciences, Division of Cell Biology, Linköping University, Linköping 58185, Sweden

**Keywords:** Head and neck cancer, Hypoxia, Radiotherapy, Biomarker, Prognostic factor, KIF14

## Abstract

Head and neck squamous cell carcinoma (HNSCC) is a disease associated with a severe mortality and high risk of distant metastasis and local recurrence. Currently, surgery and radiotherapy are the main treatment modes, however, therapeutic efficacy of radiotherapy is linked to tumor resistance. Hypoxia has been shown to affect outcome of radiotherapy in HNSCC patients. The aim of this study was to verify the expression of the previously identified hypoxia-responsive genes (*CA9, CASP14, LOX, GLUT3, SERPINE1, AREG, EREG, CCNB1* and *KIF14*) in HNSCC patient material as well as assess their prognostic potential. Tumor biopsies obtained before start of radiotherapy from 32 HNSCC patients classified as responders or non-responders were investigated in this study. The mRNA expression was quantified using RT-qPCR. The mRNA expression of *CA9, SERPINE1* and *KIF14* was significantly higher in the analyzed patient material compared with the non-cancerous oral tissue. Moreover, the *KIF14* mRNA expression was significantly higher in the responder group compared to non-responders. Further studies demonstrated that knockdown of KIF14 reverses its radiosensitizing capability. Additionally, low expression of *KIF14* mRNA correlated with significantly shorter OS (overall survival). In conclusion, our results suggest that *KIF14* might be a useful prognostic and predictive marker in HNSCC.

## Highlights

• The mRNA expression of hypoxia-responsive genes is head and neck squamous cell carcinoma (HNSCC)-specific.
• *KIF14* mRNA is markedly altered in the group of patients responding to radiotherapy, which was reflected in the clinical outcome of HNSCC patients.
• Silencing of KIF14 reverses its radiosensitizing capability.

## Introduction

Head and neck cancer is the seventh most common cancer worldwide [1]. 90 % of head and neck cancers are squamous cell carcinomas (HNSCC) that originate from the oral cavity, oropharynx, larynx, hypopharynx, and nasopharynx. The major risk factors behind HNSCC are use of tobacco, alcohol, and exposure to high-risk human papilloma virus. The treatment modalities of HNSCC depends on the TNM stage and primary tumor site [1,2]. A single modality treatment, surgery or radiotherapy is used for early-stage tumor. Advanced stages of the disease require multimodal treatment based on radiotherapy combined with cisplatin-based chemotherapy. Despite the progress in treatment strategies, advanced HNSCC still yields a crude prognosis (5-year overall survival, <50 %), carries a high risk of distant metastasis and local recurrence [3]. Therapy failure is often associated with radioresistance and its effectiveness is greatly attenuated by hypoxia within the tumoral tissue [4,5].

HNSCC has shown significantly worse outcome in hypoxic tumors compared to tumors expressing less hypoxic features [6]. The importance of hypoxia-induced radioresistance has been acknowledged for decades pointing at complexity of interaction between tumor hypoxia and radiotherapy in the search for radiosensitizing agents [4]. Furthermore, hypoxic tumors have shown to exhibit aggressive tumor phenotypes with increased risk of progression, metastasis, and recurrence [7,8]. To combat the hypoxic state, cells have acquired several adaptive mechanisms, in which hypoxia inducible factor (HIF) pathway is central. Hypoxic tumor microenvironment is a non-cellular factor that induces epithelial-mesenchymal transition (EMT) by HIF-1-mediated activation of Twist or Snail that are important drivers of EMT [9]. EMT serves as one of the key factors affecting tumor cell proliferation, invasion and metastasis, angiogenesis or even cancer-stem cell phenotype of cancer cells [10]. Hypoxia is believed to have impact on cancer stem cell (CSC) phenotype and leads to dysregulation of cancer stemness transcription factors such as Sox2 and Nanog [11].

Moreover, HIF mediates the transcription of several hundreds of genes implicated in cell division, angiogenesis, and cell metabolism as well as regulates expression of CA9 [12]. CA9 has been associated with hypoxia and poor prognosis in a range of tumor types [13,14]. In HNSCC, hypoxia is often correlated with changes in cellular metabolism and responses to radio- and chemotherapy. Moreover, genes responsible for glucose metabolism (GLUT1 and GLUT3) and angiogenesis (VEGF) are also activated by tumor hypoxia [15,16].

In our previous study [17], hypoxia was found to induce an increased survival capacity after radiation and an increase expression of EMT- and CSC-associated genes in HNSCC cell lines. Furthermore, a microarray analysis identified a panel of hypoxia-responsive genes and among genes highly upregulated by hypoxia were *CA9, CASP14, LOX, GLUT3, SERPINE1* and highly downregulated *AREG, EREG, CCNB1* and *KIF14*. Moreover, further network analysis has revealed the involvement of the microarray-identified genes in cancer, cell death and survival as well as cellular assembly and organization.

In the present study we further evaluated the impact of the above-mentioned genes in 32 HNSCC biopsies from patients treated with radiotherapy on treatment response and overall survival.

## Material and methods

### Patient material and clinicopathological data

The study material included 32 fresh-frozen, biopsy specimens obtained before initiation of radiotherapy from patients diagnosed with HNSCC. The use of patient material has been approved by the Ethical Committee of Linköping. The biopsies were collected between the years of 2003 and 2009. All patients received radiotherapy as accordingly: 13 (40.6 %) solely received radiotherapy, 15 (46.9 %) preoperative radiotherapy, 3 (9.4 %) postoperative and 1 (3.1 %) received chemotherapy in addition to radiotherapy and surgery. The patient group was retrospectively divided as described in our preceding study [18,19] into responder and non-responder according to therapeutic response. If the tumor size was reduced during radiotherapy and no recurrent disease was observed with within 1 year following radiotherapy, a patient was considered a responder. A patient was considered a non-responder if the tumor grew during ongoing radiation treatment or patients had a relapse of disease within 6 months of radiation treatment. Within the above-mentioned panel of 32 HNSCC patients included in the analysis, 16 patients were identified as non-responders and 16 as responders (Table 1).

**Table 1:** Clinical characteristics of patients included in the study.

Characteristics	Non responder N ( %) (total=16)	Responder N ( %) (total=16)
Sex		
Man	10 (62.5)	6 (37.5)
Woman	6 (37.5)	10 (62.5)
Age (years)		
Mean value	67	67.7
Range	37–86	55–81
Primary tumor site		
Gingiva	3 (18.8)	3 (18.8)
Larynx	4 (25)	3 (18.8)
Tongue	6 (37.5)	5 (31.3)
Trigonum retromolare	1 (6.3)	1 (6.3)
Buccal mucosa	2 (12.5)	1 (6.3)
Hypopharynx	0 (0)	1 (6.3)
Palate mucosa	0 (0)	1 (6.3)
Floor of mouth	0 (0)	1 (6.3)
Primary tumor (T)		
T2	6 (37.5)	8 (50)
T3	6 (37.5)	4 (25)
T4	4 (25)	3 (18.8)
Undetermined	0 (0)	1 (6.3)
Regional lymph nodes (N)		
N0	10 (62.5)	11 (68.8)
N1	2 (12.5)	0 (0)
N2	3 (18.8)	4 (25)
Undetermined	1 (6.3)	1 (6.3)
Distant metastasis (M)		
M0	16 (100)	15 (93.8)
Undetermined	0 (0)	1(6.3)
Differentiation grade		
High	5 (31.3)	2–3 (12.5–18.8)
Moderate	6–8 (37.5–50)	10–11 (62.5–68.8)
Low	3–5 (18.8–31.3)	3 (18.8)

The non-responder group consisted of 3 (18.8 %) samples from the gingiva, 4 (25 %) from the larynx, 6 (37.5 %) from the tongue, 1 (6.3 %) from the trigonum retromolare and 2 (12.5 %) from the buccal mucosa. The responder group consisted of 3 (18.8 %) samples from the gingiva, 3 (18.8 %) from the larynx, 5 (31.3) from the tongue, 1 (6.3 %) from the trigonum retromolare, 1 (6.3 %) from the buccal mucosa, 1 (6.3 %) from the hypopharynx, 1 (6.3 %) from the mucosa of the palate and 1 (6.3 %) from the floor of the mouth. Patient characteristics, primary tumor site, degree of differentiation and TNM staging are shown in Table 1. Non-cancerous oral tissues, collected from the sites as far as possible from the tumor margin were included in the study as controls.

### Cell culture

HNSCC cell lines (LK0412, LK0532, LK0824, LK0827, LK0850, LK0863, LK0902, LK0927 and LK0949), were cultured in Keratinocyte-SFM medium containing 10 % fetal bovine serum, 50 U/ml and streptomycin 50 U/ml streptomycin (all from GIBCO, Invitrogen Corporation, Paisly, UK) in 37 °C incubator with 5 % CO_2_. The media was changed twice a week, and cell lines were subcultured weekly after detachment with 0.25 % trypsin + 0.02 % EDTA. The cultures were routinely checked for mycoplasma contamination using the MycoAlert Mycoplasma Detection Kit (Lonza, Walkersville, MD USA).

### RNA extraction

Total RNA extraction from frozen tissues was performed using the AllPrep DNA/RNA/miRNA Kit (Qiagen, Hilden, Germany) according to manufacturer's instructions. Tissues were disrupted and homogenized in RLT buffer containing β-marcaptoethanol and one stainless steel bead using TissueLyser II instrument. The RNA fraction was separated using RNeasy Mini spin columns. RNA was dissolved in RNase-free water and stored at −70 °C. RNA concentration was measured using NanoDrop (TermoFisher, Massachusetts, USA).

### cDNA synthesis and RT-qPCR

RNA was converted to cDNA using the High-Capacity RNA-to-cDNA kit (Applied Biosystems, Stockholm, Sweden). The RT-PCR analysis was performed on a 7500 Fast Real-Time PCR system (Applied Biosystems, Waltham, MA, USA). TaqMan FAM/MGB probes: *CA9* (Assay ID:Hs00154208_m1), *SERPINE1* (Assay ID:Hs00167155_m1), *CASP14* (Assay ID:Hs00201637_m1), *LOX* (Assay ID:Hs00942480_m1), *GLUT3* (Assay ID:Hs00359840_m1), *CCNB1* (Assay ID:Hs99999188_m1), *AREG* (Assay ID:Hs00950669_m1), *EREG* (Assay ID:Hs00914313_m1), *KIF14* (Assay ID:Hs00978236_m1), *GAPDH* (Assay ID:Hs02758991_g1) and *β-actin* (Assay ID:Hs99999903_m1) were used for the RT-qPCR reaction. Amplification of GAPDH and β-actin was used as internal control. The data were calculated according to the comparative Ct method to present the data as fold differences in the expression levels relative to the control sample.

### Western blot

Whole cell extracts were prepared from the HNSCC cells using RIPA buffer for 30 min at 4 °C and the protein concentration was determined using the Bio-Rad DC Protein Assay, and 30 µg of total cell extracts was subjected to Western blotting. The membranes were incubated with anti-KIF14 antibody (1:1000; Abcam, UK), followed by a goat anti-rabbit antibody conjugated to HRP (1:5000; Santa Cruz Biotechnology, USA). The bands were visualized with the Western Blotting Luminol Reagent (Bio-Rad, USA). Equal loading was verified by reprobing the membranes with an HRP-conjugated anti-βactin antibody (1:2000; Santa Cruz Biotechnology, USA).

### RNA interference

Cells were seeded at a density of 12 000 cells/cm^2^ and were transfected 24 h later with 10 nmol/l FlexiTube siRNA (Cat. No 1,027,416; Qiagen, Germany) against KIF14 (SI02781324 and SI02781163; 1:1 ratio of the two siRNA clones was used for each transfection), or a nontargeting siRNA with no homology to any known human gene (AllStars Negative Control siRNA: 1,027,280) with the HiPerFect transfection reagent (Qiagen, Germany). Knockdown was verified by RT-qPCR and Western blot 24 h post transfection. For the cell proliferation study, cells were seeded into 12-well plates (BD Falcon, USA) and irradiated (2, 4 or 6 Gy) 48 h after transfection.

### Cell proliferation assay

Tumor cells were seeded in 12-well plates at densities of 300–800 cells/cm^2^, depending on the plating efficiency of each cell line. Selected cells were irradiated (2, 4 or 6 Gy) with 4 MeV photons generated by a linear accelerator (Clinac 4/100, Varian, Palo Alto, USA), delivering a dose-rate of 2.0 Gy/min. Nine days after treatment, cells were fixed in 4 % paraformaldehyde (20 min), stained with crystal violet solution (0.04 % in 1 % ethanol) for 20 min at room temperature and then washed and air-dried. After solubilization in 1 % SDS, the optical density at 550 nm was measured using a Victor plate reader (EG & G Wallac).

### Statistical analysis

Statistical analysis was performed using Prism 9.0 (GraphPad Software, Inc., La Jolla, CA, USA) and SPSS IBM version 27.0 (IBM Corporation, Armonk, NY, USA). The log2 transformed mRNA expression data were used in the analysis. Shapiro Wilk test was used to assess if the dataset is normally distributed. The high and low mRNA levels were assessed based on the median mRNA expression. The multiple comparison analysis was performed using one-way ANOVA followed by post-hoc Tukey's multiple correction test. The mRNA expression of the genes of interest was analyzed for correlation with overall survival of the patients using the Mantel-Cox log-rank statistics in SPSS software and presented as Kaplan-Meier survival curves depending on low or high mRNA expression. Differences between two groups (non-targeting siRNA and KIF14 siRNA) were analyzed with the unpaired Student *t*-test. All values obtained were represented as mean ± SD of at least three independent experiments. *p ≤ 0.05, ^⁎⁎^*p* ≤ 0.01, ^⁎⁎⁎^*p* ≤ 0.001, ^⁎⁎⁎⁎^*p* ≤ 0.0001.

## Results

### The expression of hypoxia-responsive genes in patients with HNSCC

In the search for potential biomarkers in HNSCC, we examined mRNA expression of five highly upregulated (*CA9, CASP14, LOX, GLUT3, SERPINE1*) and four highly downregulated (*AREG, EREG, CCNB1* and *KIF14*) hypoxia-responsive genes in 32 HNSCC tumors and six adjacent normal oral tissue. Depending on response to radiotherapy, the patients were divided into responders (*n* = 16) or non-responders (*n* = 16) group. Non-tumoral oral tissues were used as control (*n* = 6). The mRNA expression analysis of the selected genes was assessed with RT-qPCR.

**Fig. 1:**
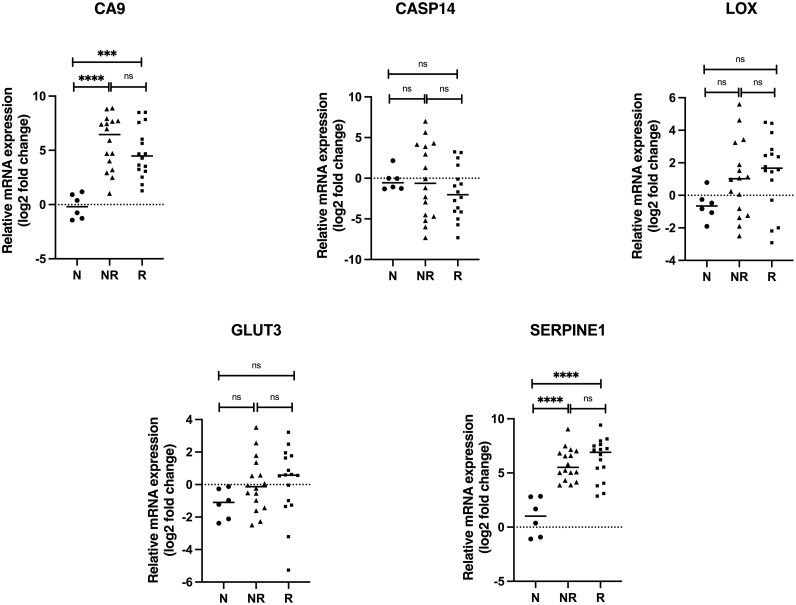
**mRNA expression of hypoxia-upregulated genes in HNSCC patients.** The mRNA expression of hypoxia-upregulated genes revealed by microarray analysis was analyzed by qRT-PCR. The log2 mRNA levels of *CA9, CASP14, LOX, GLUT3* and *SERPINE1* in tumor tissue are shown relative to the expression in normal oral tissue. Data are shown as mean values ± SD; *n* = 3. *p ≤ 0.05, ^⁎⁎^*p* ≤ 0.01, ^⁎⁎⁎^*p* ≤ 0.001, ^⁎⁎⁎⁎^*p* ≤ 0.0001. Abbreviations: N, adjacent normal oral tissue; NR, non-responder; R, responder.

Our results show a significantly higher mRNA expression of the hypoxia marker *CA9* and *SERPINE1* in all tumor biopsies (responders and non-responders) compared to normal tissue. Although *CA9* mRNA expression was lower in the responder group in comparison to non-responder, the difference did not reach significance in the analyzed patient material. The *SERPINE1* mRNA expression was slightly higher in responder group, however this difference was not significant. The mRNA expression of other highly upregulated genes (*LOX* and *GLUT3*) in hypoxic HNSCC cell lines was higher in HNSCC patients compared to normal tissues but the differences were not statistically significant. The mRNA expression of *CASP14*, highly upregulated gene in hypoxic HNSCC cell lines remained at similar level when compared non-tumoral and tumoral tissue. There were no significant differences regarding mRNA expression of analyzed hypoxia-upregulated genes between responder and non-responder groups (Fig. 1).

Regarding the hypoxia-downregulated genes, the mRNA expression level of *AREG, CCNB1* and *KIF14* did not demonstrate the same pattern as in hypoxic HNSCC cell lines. In turn, we observed higher expression of *AREG* mRNA in HNSCC patients with trend to higher expression in the responder group. Like *AREG, KIF14* mRNA level was higher in HNSCC samples than that in control group. Interestingly, the responder group exhibited significantly higher level of *KIF14* mRNA compared to the non-responder group.

**Fig. 2:**
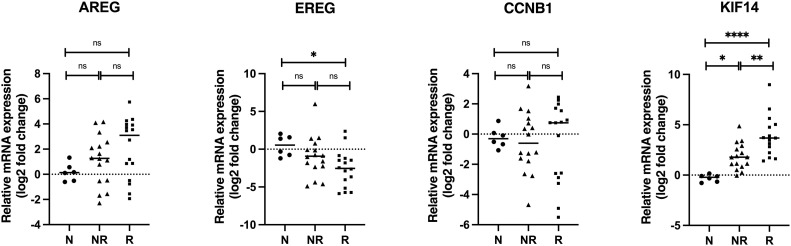
**mRNA expression of hypoxia-downregulated genes in HNSCC patients.** The mRNA expression of hypoxia-downregulated genes revealed by microarray analysis was analyzed by qRT-PCR. The log2 mRNA levels of *AREG, EREG, CCNB1* and *KIF14* in tumor tissue are shown relative to the expression in normal oral tissue. Data are shown as mean values ± SD; *n* = 3. *p ≤ 0.05, ^⁎⁎^*p* ≤ 0.01, ^⁎⁎⁎^*p* ≤ 0.001, ^⁎⁎⁎⁎^*p* ≤ 0.0001. Abbreviations: N, adjacent normal oral tissue; NR, non-responder; R, responder.

The *EREG* mRNA expression was lower in both responder and non-responder group compared to normal tissue, but its expression differed significantly between the responder and control group. The *CCNB1* mRNA expression in HNSCC samples did not deviate much from non-tumoral tissue with slight but not significant increase in the responder group (Fig. 2).

### The impact of the candidate hypoxia-responsive genes on overall survival in HNSCC

**Fig. 3:**
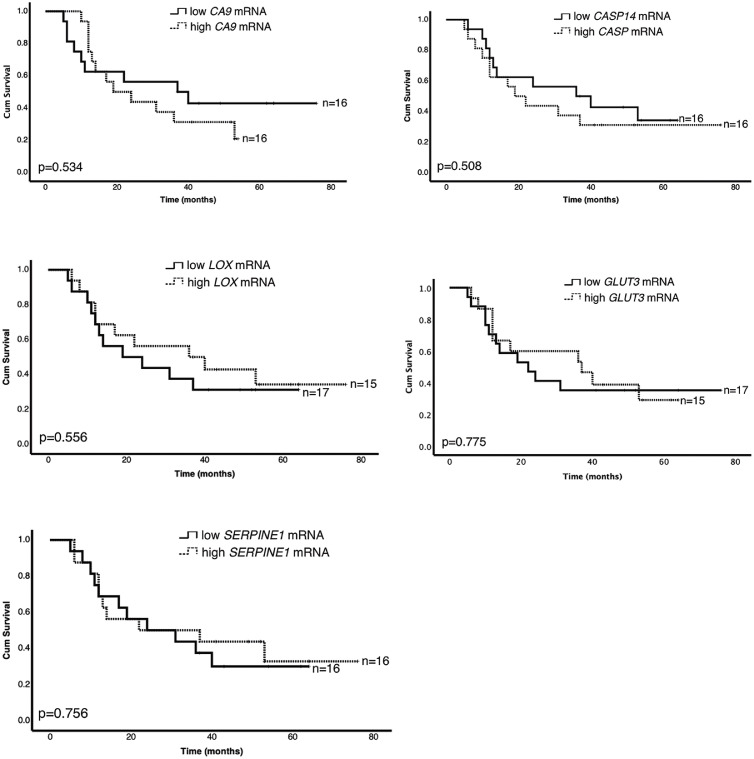
**Prognostic significance of hypoxia-upregulated genes in HNSCC patients.** Kaplan-Meier log-rank survival analysis of HNSCC patients regarding different mRNA expression of previously identified, hypoxia-upregulated genes.

**Fig. 4:**
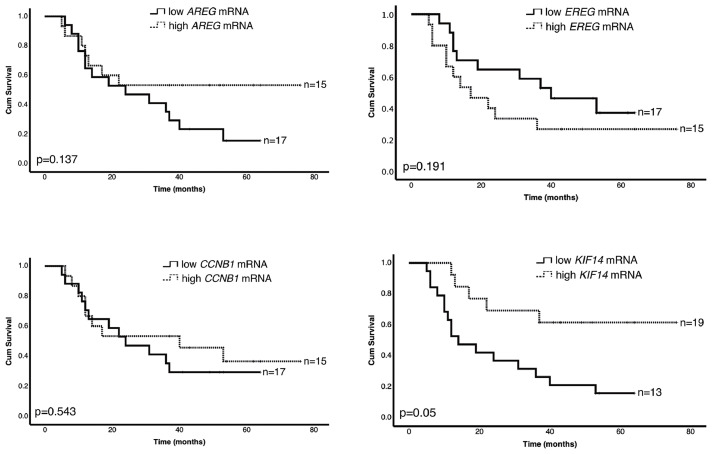
**Prognostic significance of hypoxia-downregulated genes in HNSCC patients.** Kaplan-Meier log-rank survival analysis of HNSCC patients regarding different mRNA expression of previously identified, hypoxia-downregulated genes revealed by microarray analysis.

**Fig. 5:**
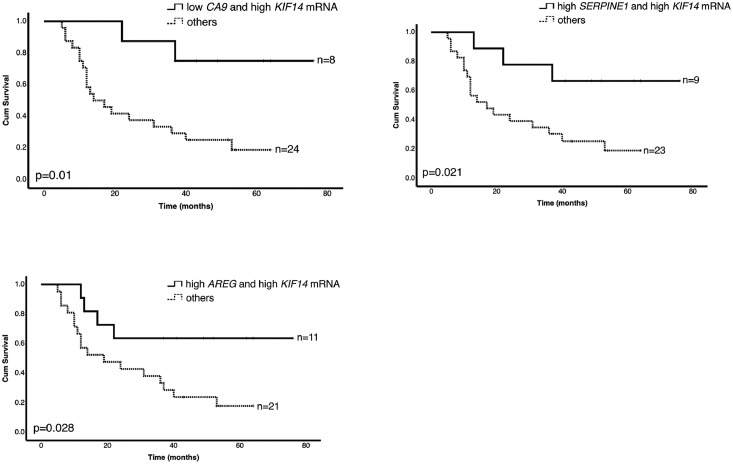
**Prognostic significance of hypoxia-responsive genes in HNSCC patients.** Kaplan Meier log-rank survival analysis of HNSCC patients regarding different mRNA expression of combined *CA9*/*KIF14, SERPINE1/KIF14 and AREG/KIF14* genes.

We further evaluated the association of hypoxia-responsive genes with overall survival of the HNSCC patients. The obtained relative mRNA expression level was divided into low and high mRNA expression. The median value for log2 fold change was used as a cut-off, where values > median were considered as high mRNA expression and values < median were considered as low mRNA expression. We observed no statistically significant differences between low and high mRNA expression regarding the analyzed hypoxia-upregulated genes (Fig. 3). Regarding the hypoxia-downregulated genes (Fig. 4), we demonstrated that patients with high *KIF14* mRNA expression showed significantly longer survival rate than patients with low *KIF14* mRNA expression (*p* = 0.05). Interestingly, simultaneous high mRNA expression of *KIF14* and low mRNA expression of *CA9* correlated with noticeably better overall survival of HNSCC patients (*p* = 0.01; Fig. 5). Furthermore, the overall survival of patients with simultaneous high *SERPINE1* and *KIF14* mRNA (*p* = 0.021) as well as high *AREG* and *KIF14* mRNA (*p* = 0.028) was better than patients with the opposite expression pattern (Fig. 5).

### The expression of KIF14 in HNSCC

Since *KIF14* mRNA expression was significantly higher in the analyzed group of HNSCC patients responding to radiotherapy, we next evaluated the expression of KIF14 on the mRNA and protein level in HNSCC cell lines that were classified as radioresistant or radiosensitive. Four radioresistant HNSCC cell lines (LK0532, LK0824, LK0827, LK0927) and five radiosensitive HNSCC cell lines (LK0412, LK0850, LK0863, LK0902, LK0949) were included in the study based on their previously established intrinsic radiosensitivity (IR) [20].

**Fig. 6:**
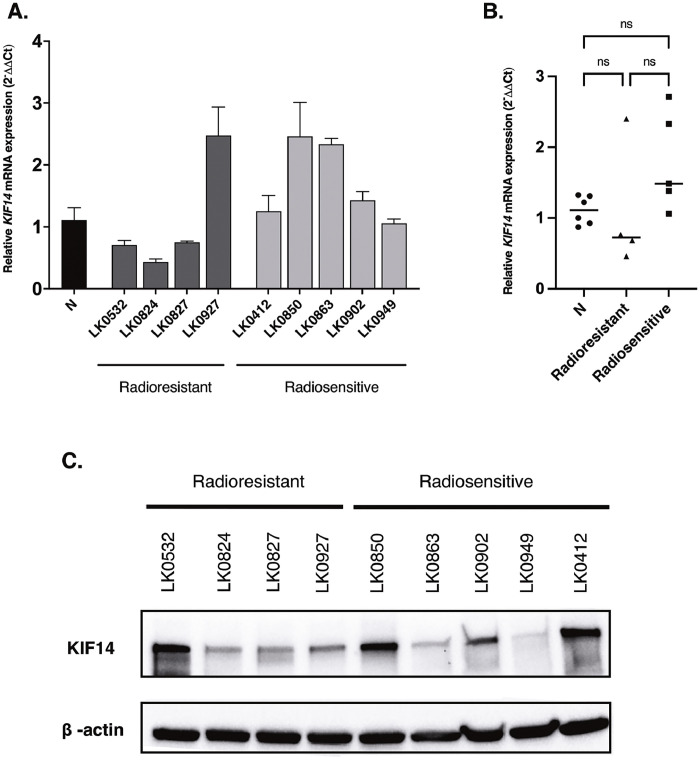
**The expression of KIF14 in HNSCC cell lines.** The relative *KIF14* mRNA expression in each individual cell line (**A**) and combined *KIF14* mRNA expression in normal oral tissue (N), radioresistant and radiosensitive cell lines (**B**). The mRNA levels are shown relative to the mRNA expression in normal oral tissue. Data are shown as means ± SD from 3 independent experiments (*n* = 3). Statistical analysis was performed using unpaired *t*-test**.** Western blot analysis of KIF14 expression in radioresistant and radiosensitive HNSCC cell lines (**C**); ns=not significant.

Our results show that higher expression of KIF14 revealed by RT-qPCR (Fig. 6A and 6B) and Western blot (Fig. 6C) analysis is not restricted to sensitive cell lines as compared to patient material. However, we observed a trend toward higher expression of *KIF14* mRNA in the radiosensitive HNSCC cell lines.

### The impact of KIF14 on radiotherapy response in HNSCC

**Fig. 7:**
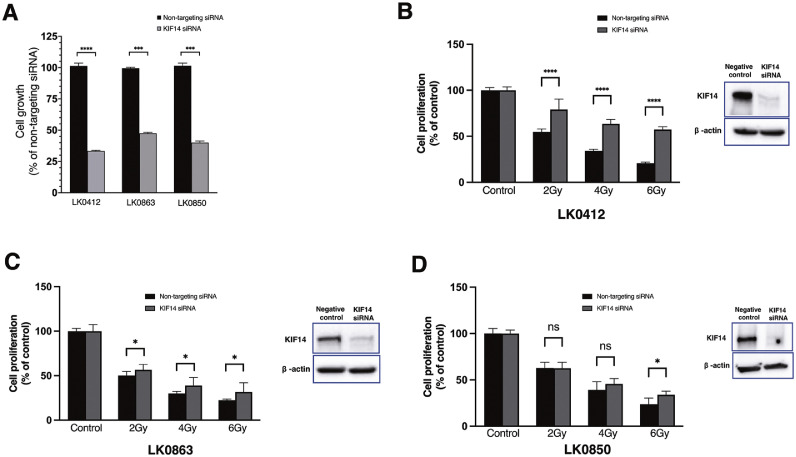
**The effect of KIF14 silencing on radiotherapy response in HNSCC cell lines.** Crystal violet assay was used to assess the proliferative ability of irradiated HNSCC cells (LK0412, LK0863 and LK0850) in the presence of *KIF14* siRNA. Following downregulation of KIF14, the growth rate of the analyzed HNSCC cell lines was measured and presented as % of control siRNA (non-targeting siRNA) **(A)**. Cell proliferation of the irradiated cells in the presence of *KIF14* siRNA is presented as the percentage of the untreated controls, and data are presented as the mean ± SD from three independent experiments performed in triplicate. Western blot analysis of knockdown efficacy of KIF14 in the analyzed cell lines is presented next to the cell proliferation analysis **(B-D)**. Statistical analysis was performed using unpaired Student *t*-test**.** *p ≤ 0.05, ^⁎⁎⁎^*p* ≤ 0.001, ^⁎⁎⁎⁎^*p* ≤ 0.0001, ns=not significant.

To explore the potential radiosensitizing role of *KIF14*, we implemented siRNA-mediated downregulation of *KIF14* and evaluated its effect on radiotherapy response in the radiosensitive cell lines. We observed a significant decrease in HNSCC cell growth after downregulation of KIF14 compared to control cells (Fig. 7A). Moreover, silencing of *KIF14* was associated with decreased sensitivity towards irradiation in the analysed radiosensitive HNSCC cell lines (Fig. 7B-D).

## Discussion

Treatment modalities for HNSCC are heavily based on radiotherapy combined with chemotherapy or surgery. Given the everyday functions in the head and neck region, the consequences of HNSCC and its treatment have a large impact on health-related quality of life [21]. The choice of initial treatment, administration, and sequencing involves consideration of toxic effects, morbidity, and preservation of function [22]. Identifying biomarkers that predict the response to radiotherapy may assist the choice of most effective treatment for the patient.

The response to radiation therapy is greatly hindered by hypoxia [4,17]. We have recently identified a panel of hypoxia-responsive genes in HNSCC cell lines that might have impact on prognosis and therapy response in HNSCC patients [17]. One of the highly upregulated genes in hypoxic HNSCC cells as well as analyzed patient material was *CA9* [23,24]. CA9 has been shown to be upregulated at both mRNA level and protein level in hypoxic tumors and correlates with poor prognosis in various types of cancer [13,25]. Hypoxia has also been shown to negatively affect radiotherapy and chemotherapy in HNSCC patients [17,[26], [27], [28]]. Our results show a distinct increase in *CA9* mRNA expression in HNSCC patients compared to non-tumoral tissues. The responder group exhibited markedly lower expression of *CA9* mRNA than non-responder group, which might be associated with the radiotherapy response. Despite this, *CA9* mRNA expression alone was not shown to be a predictor of overall survival in the analyzed patient material. In another study [29] involving 39 HNSCC samples, CA9 expression did not have a significant impact on overall survival but on local relapse free survival (LRFS).

Another highly hypoxia-responsive gene in HNSCC cell lines, namely *SERPINE1*showed to be upregulated in HNSCC patients. High expression of *SERPINE1* has been associated with poor prognosis in head and neck patients [30]. Moreover, SERPINE1 expression is involved in epithelial to mesenchymal transition (EMT), gain of stem cell properties and resistance to anti-cancer therapy in head and neck cancer [31]. In our study, expression of *SERPINE1* mRNA did not show to be independent prognostic marker, however high expression of *SERPINE1* mRNA in combination with high *KIF14* mRNA expression correlated with better overall survival in HNSCC. This observation points at potential interconnection between SERPINE1 and KIF14 that warrants further investigation.

The hypoxia-induced mRNA expression of *CASP14, GLUT3* and *LOX* in HNSCC cell lines was partially reflected in the analyzed patient cohort. These genes have been shown to drive carcinogenesis in many types of cancer [32], [33], [34], [35]. Upregulation of the *CASP14* as a novel hypoxia-regulated gene bound by HIF1 has previously been observed in breast cancer [36] and our previous study in HNSCC cell lines has pointed at *CASP14* as highly hypoxia-upregulated. Although hypoxia-specific marker *CA9* was significantly higher in the analyzed patient material, *CASP14* mRNA seemed not to be hypoxia-dependent. Such discrepancy might be a result of different signaling between 2D-grown HNSCC cells and 3D tumor mass. Moreover, our patient cohort is too small to draw conclusion from current observation.

Hypoxia has also been shown to upregulate *LOX* expression thereby promoting invasion and metastasis of breast and ovarian cancer cell lines [37,38]. Moreover, hypoxia leads to increase of glucose uptake by upregulating GLUT1 and GLUT3 and GLUT3 expression correlates with poor prognosis in various cancers including oral squamous cell carcinoma. The trend towards higher mRNA expression of *LOX* and *GLUT3* in HNSCC patients was observed, however no correlation with prognosis of the HNSCC patients regarding the mRNA expression of these genes was noted [39], [40], [41], [42], [43].

Among the genes downregulated by hypoxia in HNSCC cell lines, a decreased mRNA expression level of *EREG* was also detected in the analyzed HNSCC patient material. EREG serves as a ligand of the epidermal growth factor receptor (EGFR) and its deregulation in different types of cancer leads to activation of EGFR signaling pathways and tumorigenesis [44]. EREG has also shown to be involved in reprogramming of cancer associated fibroblasts (CAF) and EMT induction in oral squamous cell carcinoma [45]. The results from *CCNB1* mRNA analysis in HNSCC patients differ from the results observed from HNSCC cell lines. Deregulation of this mitosis-linked molecule has been shown in various types of cancer [46], [47], [48]. In our study, we did not observe significant differences between *CCNB1* mRNA expression in non-tumoral and tumoral tissue, specifically in the non-responder group. Hypoxia itself is associated with resistance to radiotherapy and hypoxia-mediated downregulation of *CCNB1* could support this process. In another study, overexpression of CCNB1 in HNSCC tumors has been shown as a marker of resistance to radiotherapy and the risk of locoregional recurrence and metastasis in patients treated with radiotherapy [49]. Whether expression of *CCNB1* and its potential prognostic significance is hypoxia-specific must be analyzed in a bigger patient cohort.

Regarding the hypoxia-downregulated genes, these genes were highly expressed in the analyzed patient cohort. Like EREG, AREG is a ligand of the EGFR and in HNSCC high expression of AREG was associated with better overall survival and progression free survival as well as greater benefit from cetuximab combined with chemotherapy [50]. Noteworthily, high expression of *AREG* mRNA in combination with high expression of *KIF14* mRNA correlated with better overall survival in the analyzed patient cohort. Interestingly, *KIF14* mRNA expression was significantly higher in the responder group that might be beneficial regarding the response to radiation therapy. KIF14 belongs to the kinesin-3 family of proteins that are involved in various processes including vesicle transport, chromosome segregation, mitotic spindle formation, and cytokinesis [51,52]. There are many reports showing oncogenic nature of KIF14 and its correlation with poor prognosis [53,54]. Overexpression of *KIF14* mRNA has been observed in several types of cancer including oral cancer [55], [56], [57], [58], [59], [60]. On the other hand, many reports point at tumor-suppressive role of KIF14 in some tumors [61,62]. In our study, patients with high *KIF14* mRNA expression had longer overall survival than those with low *KIF14* mRNA expression. Like our results, low expression of *KIF14* mRNA has been related to worse overall survival in lung adenocarcinoma and colorectal cancer [61,63]. Interestingly, the prognostic value of *KIF14* mRNA expression in HNSCC patients was significantly increased by simultaneous high mRNA expression of *KIF14* and low mRNA expression of *CA9.* CA9 is highly related to hypoxia and high levels of CA9 might accelerate survival of cancer cells and negatively affect response to treatment. It has been reported that high CA9 expression is associated with reduced overall survival in oral cavity squamous cell carcinoma and poor pathological T-stage [64,65]. Increased expression of *KIF14* mRNA was also observed in the responder group suggesting a predictive role of *KIF14* in radiotherapy response. Radiotherapy treatment is known to be beneficial for rapidly dividing cells and KIF14 is involved in many biological functions including proliferation. High *KIF14* mRNA expression in responder group seems to be beneficial and might indicate sensitization of tumor cells to radiotherapy. Moreover, only patients with low CA9 mRNA and high *KIF14* mRNA expression showed better overall survival pointing at hypoxia as regulator of *KIF14*.

Regarding the fact that patients responding to radiotherapy have higher mRNA expression of *KIF14* than non-responders, we next determined the potential role of KIF14 in radiotherapy response. We show that downregulation of KIF14 leads to inhibition of cell proliferation and at the same time cells with lower expression of KIF14 become more resistant to radiotherapy. The possible explanation could be that ionizing radiation is based on the premise that rapidly proliferating cancer cells are more sensitive to DNA damage compared to cells with slow proliferation rate.

## Conclusions

In conclusion, our results indicate that the pattern of hypoxia-responsive genes in HNSCC cell lines is HNSCC tumor-specific. We show also that mRNA expression of *KIF14* is a potential diagnostic marker and might serve as a predictor of radiation treatment response in HNSCC. However, more studies using larger patient cohort are required to study the hypoxia-associated expression signatures and their utility in HNSCC diagnosis and treatment. Additionally, a more mechanistic insight into KIF14 in HNSCC biology and the implication for radiotherapy are needed.

## Data availability

The data can be obtained upon a reasonable request from the corresponding author.

## Ethical approval statement

The study was approved by the Research Ethics Committee of the Linköping University (approval no. 03–537). Consent for publication was obtained from all patients.

## CRediT authorship contribution statement

**Natasa Matic:** Data curation, Methodology, Writing – original draft. **Lina Pettersson:** Data curation, Methodology, Writing – original draft. **Felicia Sellebjerg:** Data curation, Methodology. **Lina Lindberg:** Data curation, Methodology. **Karin Roberg:** Conceptualization, Supervision, Funding acquisition, Writing – review & editing. **Emilia Wiechec:** Conceptualization, Methodology, Data curation, Supervision, Writing – original draft, Writing – review & editing.

## Declaration of Competing Interest

The authors declare that they have no known competing financial interests or personal relationships that could have appeared to influence the work reported in this paper.

## References

[1] Mody M.D., Rocco J.W., Yom S.S., Haddad R.I., Saba N.F. (2021). Head and neck cancer. Lancet.

[2] Johnson D.E., Burtness B., Leemans C.R., Lui V.W.Y., Bauman J.E., Grandis J.R. (2020). Head and neck squamous cell carcinoma. Nat. Rev. Dis. Primers.

[3] Pulte D., Brenner H. (2010). Changes in survival in head and neck cancers in the late 20th and early 21st century: a period analysis. Oncologist.

[4] Telarovic I., Wenger R.H., Pruschy M. (2021). Interfering with tumor hypoxia for radiotherapy optimization. J. Exp. Clin. Cancer Res..

[5] Perri F., Pacelli R., Della Vittoria Scarpati G., Cella L., Giuliano M., Caponigro F., Pepe S. (2015). Radioresistance in head and neck squamous cell carcinoma: biological bases and therapeutic implications. Head Neck.

[6] Overgaard J. (2007). Hypoxic radiosensitization: adored and ignored. J. Clin. Oncol..

[7] Muz B., de la Puente P., Azab F., Azab A.K. (2015). The role of hypoxia in cancer progression, angiogenesis, metastasis, and resistance to therapy. Hypoxia (Auckl).

[8] Fares J., Fares M.Y., Khachfe H.H., Salhab H.A., Fares Y. (2020). Molecular principles of metastasis: a hallmark of cancer revisited. Signal. Transduct. Target. Ther..

[9] Qiao L., Chen Y., Liang N., Xie J., Deng G., Chen F., Wang X., Liu F., Li Y., Zhang J. (2022). Targeting epithelial-to-mesenchymal transition in radioresistance: crosslinked mechanisms and strategies. Front. Oncol..

[10] Nantajit D., Lin D., Li J.J. (2015). The network of epithelial-mesenchymal transition: potential new targets for tumor resistance. J. Cancer Res. Clin. Oncol..

[11] Schulz A., Meyer F., Dubrovska A., Borgmann K. (2019). Cancer stem cells and radioresistance: DNA repair and beyond. Cancers (Basel).

[12] Jun J.C., Rathore A., Younas H., Gilkes D., Polotsky V.Y. (2017). Hypoxia-Inducible factors and cancer. Curr. Sleep Med. Rep..

[13] Tanaka N., Kato H., Inose T., Kimura H., Faried A., Sohda M., Nakajima M., Fukai Y., Miyazaki T., Masuda N., Fukuchi M., Kuwano H. (2008). Expression of carbonic anhydrase 9, a potential intrinsic marker of hypoxia, is associated with poor prognosis in oesophageal squamous cell carcinoma. Br. J. Cancer.

[14] Giatromanolaki A., Koukourakis M.I., Sivridis E., Pastorek J., Wykoff C.C., Gatter K.C., Harris A.L. (2001). Expression of hypoxia-inducible carbonic anhydrase-9 relates to angiogenic pathways and independently to poor outcome in non-small cell lung cancer. Cancer Res..

[15] Choudhry H., Harris A.L. (2018). Advances in hypoxia-inducible factor biology. Cell Metab..

[16] Lv X., Li J., Zhang C., Hu T., Li S., He S., Yan H., Tan Y., Lei M., Wen M., Zuo J. (2017). The role of hypoxia-inducible factors in tumor angiogenesis and cell metabolism. Genes Dis..

[17] Wiechec E., Matic N., Ali A., Roberg K. (2022). Hypoxia induces radioresistance, epithelialmesenchymal transition, cancer stem celllike phenotype and changes in genes possessing multiple biological functions in head and neck squamous cell carcinoma. Oncol. Rep..

[18] Farnebo L., Tiefenböck K., Ansell A., Thunell L.K., Garvin S., Roberg K. (2013). Strong expression of survivin is associated with positive response to radiotherapy and improved overall survival in head and neck squamous cell carcinoma patients. Int. J. Cancer.

[19] Wiechec E., Magan M., Matic N., Ansell-Schultz A., Kankainen M., Monni O., Johansson A.C., Roberg K. (2021). Cancer-Associated fibroblasts modulate transcriptional signatures involved in proliferation, differentiation and metastasis in head and neck squamous cell carcinoma. Cancers (Basel).

[20] Jedlinski A., Ansell A., Johansson A.C., Roberg K. (2013). EGFR status and EGFR ligand expression influence the treatment response of head and neck cancer cell lines. J. Oral Pathol. Med..

[21] Johnson D.E., Burtness B., Leemans C.R., Lui V.W.Y., Bauman J.E., Grandis J.R. (2020). Head and neck squamous cell carcinoma. Nature Rev. Dis. Primers.

[22] Chow L.Q.M. (2020). Head and neck cancer. New England J; Med;.

[23] Kaluz S., Kaluzova M., Liao S.Y., Lerman M., Stanbridge E.J. (2009). Transcriptional control of the tumor- and hypoxia-marker carbonic anhydrase 9: a one transcription factor (HIF-1) show?. Biochim. Biophys. Acta.

[24] Pastorekova S., Gillies R.J. (2019). The role of carbonic anhydrase IX in cancer development: links to hypoxia, acidosis, and beyond. Cancer Metastasis Rev..

[25] Wykoff C.C., Beasley N.J.P., Watson P.H., Turner K.J., Pastorek J., Sibtain A., Wilson G.D., Turley H., Talks K.L., Maxwell P.H., Pugh C.W., Ratcliffe P.J., Harris A.L. (2000). Hypoxia-Inducible expression of tumor-associated carbonic anhydrases. Cancer Res..

[26] Hong B., Lui V.W., Hashiguchi M., Hui E.P., Chan A.T. (2013). Targeting tumor hypoxia in nasopharyngeal carcinoma. Head Neck.

[27] Wildeman M.A., Gibcus J.H., Hauptmann M., Begg A.C., van Velthuysen M.L., Hoebers F.J., Mastik M.F., Schuuring E., van der Wal J.E., van den Brekel M.W. (2009). Radiotherapy in laryngeal carcinoma: can a panel of 13 markers predict response?. Laryngoscope.

[28] Koukourakis M.I., Giatromanolaki A., Sivridis E., Simopoulos K., Pastorek J., Wykoff C.C., Gatter K.C., Harris A.L. (2001). Hypoxia-regulated carbonic anhydrase-9 (CA9) relates to poor vascularization and resistance of squamous cell head and neck cancer to chemoradiotherapy. Clin. Cancer Res..

[29] Koukourakis M.I., Giatromanolaki A., Danielidis V., Sivridis E. (2008). Hypoxia inducible factor (HIf1alpha and HIF2alpha) and carbonic anhydrase 9 (CA9) expression and response of head-neck cancer to hypofractionated and accelerated radiotherapy. Int. J. Radiat. Biol..

[30] Pavon M.A., Arroyo-Solera I., Tellez-Gabriel M., Leon X., Viros D., Lopez M., Gallardo A., Cespedes M.V., Casanova I., Lopez-Pousa A., Mangues M.A., Quer M., Barnadas A., Mangues R. (2015). Enhanced cell migration and apoptosis resistance may underlie the association between high SERPINE1 expression and poor outcome in head and neck carcinoma patients. Oncotarget.

[31] Pavon M.A., Arroyo-Solera I., Cespedes M.V., Casanova I., Leon X., Mangues R. (2016). uPA/uPAR and SERPINE1 in head and neck cancer: role in tumor resistance, metastasis, prognosis and therapy. Oncotarget.

[32] Handa T., Katayama A., Yokobori T., Yamane A., Horiguchi J., Kawabata-Iwakawa R., Rokudai S., Bao P., Gombodorj N., Altan B., Kaira K., Asao T., Kuwano H., Nishiyama M., Oyama T. (2017). Caspase14 expression is associated with triple negative phenotypes and cancer stem cell marker expression in breast cancer patients. J. Surg. Oncol..

[33] Fang H.Y., Chen C.Y., Hung M.F., Hsiao Y.T., Chiang T.C., Lin T.Y., Chang H.W., Chow K.C., Ko W.J. (2011). Caspase-14 is an anti-apoptotic protein targeting apoptosis-inducing factor in lung adenocarcinomas. Oncol. Rep..

[34] Nishioka T., Eustace A., West C. (2012). Lysyl oxidase: from basic science to future cancer treatment. Cell Struct. Funct..

[35] Ancey P.B., Contat C., Meylan E. (2018). Glucose transporters in cancer - from tumor cells to the tumor microenvironment. FEBS J..

[36] Ye I.C., Fertig E.J., DiGiacomo J.W., Considine M., Godet I., Gilkes D.M. (2018). Molecular portrait of hypoxia in breast cancer: a prognostic signature and novel HIF-regulated genes. Mol. Cancer Res..

[37] Ji F., Wang Y., Qiu L., Li S., Zhu J., Liang Z., Wan Y., Di W. (2013). Hypoxia inducible factor 1alpha-mediated LOX expression correlates with migration and invasion in epithelial ovarian cancer. Int. J. Oncol..

[38] Nagaraja G.M., Othman M., Fox B.P., Alsaber R., Pellegrino C.M., Zeng Y., Khanna R., Tamburini P., Swaroop A., Kandpal R.P. (2006). Gene expression signatures and biomarkers of noninvasive and invasive breast cancer cells: comprehensive profiles by representational difference analysis, microarrays and proteomics. Oncogene.

[39] Liu Y., Li Y.M., Tian R.F., Liu W.P., Fei Z., Long Q.F., Wang X.A., Zhang X. (2009). The expression and significance of HIF-1alpha and GLUT-3 in glioma. Brain Res..

[40] Schlosser H.A., Drebber U., Urbanski A., Haase S., Baltin C., Berlth F., Neiss S., von Bergwelt-Baildon M., Fetzner U.K., Warnecke-Eberz U., Bollschweiler E., Holscher A.H., Monig S.P., Alakus H. (2017). Glucose transporters 1, 3, 6, and 10 are expressed in gastric cancer and glucose transporter 3 is associated with UICC stage and survival. Gastric Cancer.

[41] Chen X., Lu P., Zhou S., Zhang L., Zhao J.H., Tang J.H. (2017). Predictive value of glucose transporter-1 and glucose transporter-3 for survival of cancer patients: a meta-analysis. Oncotarget.

[42] Ayala F.R., Rocha R.M., Carvalho K.C., Carvalho A.L., da Cunha I.W., Lourenco S.V., Soares F.A. (2010). GLUT1 and GLUT3 as potential prognostic markers for oral squamous cell carcinoma. Molecules.

[43] Le Q.T., Harris J., Magliocco A.M., Kong C.S., Diaz R., Shin B., Cao H., Trotti A., Erler J.T., Chung C.H., Dicker A., Pajak T.F., Giaccia A.J., Ang K.K. (2009). Validation of lysyl oxidase as a prognostic marker for metastasis and survival in head and neck squamous cell carcinoma: radiation therapy oncology group trial 90-03. J. Clin. Oncol..

[44] Cheng W.L., Feng P.H., Lee K.Y., Chen K.Y., Sun W.L., Van Hiep N., Luo C.S., Wu S.M. (2021). The role of EREG/EGFR pathway in tumor progression. Int. J. Mol. Sci..

[45] Wang Y., Jing Y., Ding L., Zhang X., Song Y., Chen S., Zhao X., Huang X., Pu Y., Wang Z., Ni Y., Hu Q. (2019). Epiregulin reprograms cancer-associated fibroblasts and facilitates oral squamous cell carcinoma invasion via JAK2-STAT3 pathway. J. Exp. Clin. Cancer Res..

[46] Yoshida T., Tanaka S., Mogi A., Shitara Y., Kuwano H. (2004). The clinical significance of Cyclin B1 and Wee1 expression in non-small-cell lung cancer. Ann. Oncol..

[47] Nozoe T., Korenaga D., Kabashima A., Ohga T., Saeki H., Sugimachi K. (2002). Significance of cyclin B1 expression as an independent prognostic indicator of patients with squamous cell carcinoma of the esophagus. Clin. Cancer Res..

[48] Kedinger V., Meulle A., Zounib O., Bonnet M.E., Gossart J.B., Benoit E., Messmer M., Shankaranarayanan P., Behr J.P., Erbacher P., Bolcato-Bellemin A.L. (2013). Sticky siRNAs targeting survivin and cyclin B1 exert an antitumoral effect on melanoma subcutaneous xenografts and lung metastases. BMC Cancer.

[49] Hassan K.A., Ang K.K., El-Naggar A.K., Story M.D., Lee J.I., Liu D., Hong W.K., Mao L. (2002). Cyclin B1 overexpression and resistance to radiotherapy in head and neck squamous cell carcinoma. Cancer Res..

[50] Kogashiwa Y., Inoue H., Kuba K., Araki R., Yasuda M., Nakahira M., Sugasawa M. (2018). Prognostic role of epiregulin/amphiregulin expression in recurrent/metastatic head and neck cancer treated with cetuximab. Head Neck.

[51] Nakagawa T., Tanaka Y., Matsuoka E., Kondo S., Okada Y., Noda Y., Kanai Y., Hirokawa N. (1997). Identification and classification of 16 new kinesin superfamily (KIF) proteins in mouse genome. Proc. Natl. Acad. Sci. USA.

[52] Carleton M., Mao M., Biery M., Warrener P., Kim S., Buser C., Marshall C.G., Fernandes C., Annis J., Linsley P.S. (2006). RNA interference-mediated silencing of mitotic kinesin KIF14 disrupts cell cycle progression and induces cytokinesis failure. Mol. Cell. Biol..

[53] Zhang Y., Yuan Y., Liang P., Zhang Z., Guo X., Xia L., Zhao Y., Shu X.S., Sun S., Ying Y., Cheng Y. (2017). Overexpression of a novel candidate oncogene KIF14 correlates with tumor progression and poor prognosis in prostate cancer. Oncotarget.

[54] Qiu H.L., Deng S.Z., Li C., Tian Z.N., Song X.Q., Yao G.D., Geng J.S. (2017). High expression of KIF14 is associated with poor prognosis in patients with epithelial ovarian cancer. Eur. Rev. Med. Pharmacol. Sci..

[55] Li K.K., Qi Y., Xia T., Chan A.K., Zhang Z.Y., Aibaidula A., Zhang R., Zhou L., Yao Y., Ng H.K. (2017). The kinesin KIF14 is overexpressed in medulloblastoma and downregulation of KIF14 suppressed tumor proliferation and induced apoptosis. Lab. Invest..

[56] Yang Z., Li C., Yan C., Li J., Yan M., Liu B., Zhu Z., Wu Y., Gu Q. (2019). KIF14 promotes tumor progression and metastasis and is an independent predictor of poor prognosis in human gastric cancer. Biochim. Biophys. Acta Mol. Basis Dis..

[57] Miyamoto I., Kasamatsu A., Yamatoji M., Nakashima D., Saito K., Higo M., Endo-Sakamoto Y., Shiiba M., Tanzawa H., Uzawa K. (2015). Kinesin family member 14 in human oral cancer: a potential biomarker for tumoral growth. Biochem. Biophys. Rep..

[58] Wang W., Shi Y., Li J., Cui W., Yang B. (2016). Up-regulation of KIF14 is a predictor of poor survival and a novel prognostic biomarker of chemoresistance to paclitaxel treatment in cervical cancer. Biosci. Rep..

[59] Corson T.W., Gallie B.L. (2006). KIF14 mRNA expression is a predictor of grade and outcome in breast cancer. Int. J. Cancer.

[60] Corson T.W., Zhu C.Q., Lau S.K., Shepherd F.A., Tsao M.S., Gallie B.L. (2007). KIF14 messenger RNA expression is independently prognostic for outcome in lung cancer. Clin. Cancer Res..

[61] Hung P.F., Hong T.M., Hsu Y.C., Chen H.Y., Chang Y.L., Wu C.T., Chang G.C., Jou Y.S., Pan S.H., Yang P.C. (2013). The motor protein KIF14 inhibits tumor growth and cancer metastasis in lung adenocarcinoma. PLoS ONE.

[62] Klimaszewska-Wisniewska A., Neska-Dlugosz I., Buchholz K., Durslewicz J., Grzanka D., Kasperska A., Antosik P., Zabrzynski J., Grzanka A., Gagat M. (2021). Prognostic Significance of KIF11 and KIF14 expression in pancreatic adenocarcinoma. Cancers (Basel).

[63] Neska-Dlugosz I., Buchholz K., Durslewicz J., Gagat M., Grzanka D., Tojek K., Klimaszewska-Wisniewska A. (2021). Prognostic impact and functional annotations of KIF11 and KIF14 expression in patients with colorectal cancer. Int. J. Mol. Sci..

[64] Brockton N.T., Klimowicz A.C., Bose P., Petrillo S.K., Konno M., Rudmik L., Dean M., Nakoneshny S.C., Matthews T.W., Chandarana S., Lau H.Y., Magliocco A.M., Dort J.C. (2012). High stromal carbonic anhydrase IX expression is associated with nodal metastasis and decreased survival in patients with surgically-treated oral cavity squamous cell carcinoma. Oral Oncol..

[65] Wang S., Fu Z., Wang Y., Sun Y., Cui L., Wang C., Liu Q., Shao D., Wang Y., Wen N. (2020). Correlation of carbonic anhydrase 9 (CA9) with pathological T-stage and prognosis in patients with oral tongue squamous cell carcinoma. Ann. Transl. Med..

